# Presentation and outcome of femoral infected non-unions in children and adolescents

**DOI:** 10.12669/pjms.37.1.3354

**Published:** 2021

**Authors:** Karim Bakhsh, Faridullah Khan Zimri, Eid Mohammad, Atiq-Ur-Rehman, Muhammad Saaiq

**Affiliations:** 1 Dr. Karim Bakhsh, FCPS. Department of Orthopedics, Bolan Medical Complex Hospital, Quetta, Pakistan; 2 Dr. Faridullah Khan Zimri, FCPS. Department of Orthopedics, NIRM, Islamabad, Pakistan; 3 Dr. Eid Mohammad, FCPS. Department of Orthopedics, Bolan Medical Complex Hospital, Quetta, Pakistan; 4 Dr. Atiq-Ur-Rehman, FCPS. Department of Orthopedics, Bolan Medical Complex Hospital, Quetta, Pakistan; 5 Dr. Muhammad Saaiq, FCPS. Department of Plastic Surgery, NIRM, Islamabad, Pakistan

**Keywords:** Ilizarov technique, Ilizarov method, Non-union, femur fractures, Road traffic accident

## Abstract

**Objectives:**

To document the presentation of infected non-unions of femur in pediatric and adolescent population and evaluate the outcome of segmental bone transport with the Ilizarov method.

**Methods:**

This prospective case series study was carried out over a period of five years, from January 01, 2015 to December 31, 2019. The study included all children and adolescent patients who presented with femoral infected non unions. The study excluded patients above the age of 16 years and those who had pathological fractures secondary to bone pathologies such as cysts, tumors or metabolic bone diseases.

**Results:**

Out of 31 patients, 27(87.09%) were males and 4(12.90%) were females. The mean age was 13.48±1.98 years. The underlying mechanisms that lead to the causation of fractures included road traffic accidents (n=23;74.19%), fall from height (n=7;22.58%) and firearm injuries (n=1;3.22%). The bone gaps ranged from 3-5 cm with a mean of 4.00± 0.856 cm. Bone union was achieved among 28(90.32%) patients. Infection was eradicated among 27(87.09%) patients whereas the remaining patients continued to suffer persistent infection. The most common complications included pin tract inflammation/ infection among (n=31;100%) patients and stiffness of knee joint among (n=19;61.29%) patients.

**Conclusion:**

Majority of the patients were males, aged 9-16 years. Road traffic accidents were the commonest cause of the fractures. The Ilizarov method of segmental bone transport was effective in treating the majority of infected non-unions.

## INTRODUCTION

Lower extremity injuries are not uncommon among children and adolescents worldwide. The open and closed femur fractures constitute one of the commonest presentations. Generally, the healing power and remodeling capacity of children is more robust than that of adults; however, the infected non-unions of femur constitute one major reason for their prolonged hospitalization as well as protracted periods of morbidity. Un-resolved bone infection is one of the leading causes of non-unions of femoral fractures among children. Common factors that contribute include: complex fractures, associated massive soft tissue trauma, de-vascularization of the bone and failure to institute appropriate and robust initial treatment at the very beginning of management.^1^-^4^

The success of the Ilizarov method is well established across a range of pediatric orthopedic conditions. There are few reported local studies regarding the use of Ilizarov method for lower limb injuries among adults, however there is scarcity of such studies regarding childhood fracture non unions.^5^,^6^

The present study was carried out to document the clinical and demographic presentation of pediatric and adolescent infected non-unions of femoral fractures and analyze the outcome of their management with Ilizarov method in terms of bone unions and complications encountered through the course of treatment.

## METHODS

This prospective case series study was carried out at the Departments of Orthopedic Surgery, National Institute of Rehabilitation Medicine (NIRM), Islamabad and Bolan Medical Complex Hospital, Quetta, Pakistan over a period of five years from January 01, 2015 to December 31, 2019. The study proceeded after approval by the hospital ethics committee. Via HEC/R/347 dated December 15, 2014. Written informed consent was taken from the parents of the patients. The study included all children and adolescent patients who presented with femoral infected non unions. The study excluded patients above the age of 16 years and those who had pathological fractures secondary to bone pathologies such as cysts, tumors or metabolic bone diseases. Patients up to 12 years of age were categorized as children whereas those aged 13-16 years were categorized as adolescents.

The patients underwent standard assessment, relevant investigations and X-rays. Tissue cultures of the infected bones were taken intraoperatively at the time of bone debridement. The clinical and demographic parameters included: gender, age, injury mechanism (such as road traffic accidents (RTAs), fall from height, level ground sports, child abuse and other causes), underlying risk factors for fractures/ non unions (such as obesity, diabetes mellitus, smoking, Vitamin-D deficiency, associated injuries sustained at the time of initial trauma, duration since injury acquisition, surgical interventions for fracture non unions already undertaken and the type of fracture. The length of bone gap following debridement, need for flap coverage of the soft tissue defects, duration of employment of the Ilizarov fixator and complications encountered through the course of management were all recorded. The AO classification system was used for classifying the femoral fractures.^7^

Under general anesthesia the surgeries were performed that entailed extensive bone debridement and application as well as removal of the Ilizarov fixator. Radiolucent traction table was used during the surgery to ensure fluoroscopic guidance for fixator application. The patient was kept in supine position and standard protocols were followed for applying the Ilizarov fixator. The same protocols were adopted while removing the fixator after successful completion of treatment. Hybrid technique was used by using a combination of half pins and K-wires. The patients were encouraged partial weight bearing and mobilization with assistive devices on the first postoperative day. Physiotherapy with range of motion exercises were encouraged for the knee, hip and ankle joints. The patients were motivated to ensure limb hygiene as well as pin tract self-care.

We defined the bone union as the presence of bridging trabeculae on three cortices, no pain on dynamization and the absent movement at the union site as confirmed on fluoroscopic examination.^5^,^6^ Once the docking was achieved, compression was done (0.25 twice weekly until union was achieved or the patient experienced pain). Dynamization was performed once radiological union was appreciated at the fracture site along with consolidation of the regenerate. The Association for the Study and Application of Methods of Ilizarov (ASAMI) criteria to employed to document, the bone results and functional results.^5^

### Statistical analysis

SPSS version 21 (SPSS Inc., Chicago, IL, USA) was used to analyse the data statistically. Descriptive statistics were employed to measure the outcomes of interest. The nominal variables were reported as frequency and percentages. The numerical data were reported as Mean ± SD. Descriptive statistics (i.e. frequency and percentages) were employed to calculate the various qualitative variables under scrutiny.

## RESULTS

Out of 31 patients, 27(87.09%) were males and 4(12.90%) were females. Their ages ranged between 9-16 years with a mean of 13.48±1.98 years. The underlying causes of fractures included RTAs (n=23;74.19%), fall from height (n=7;22.58%) and fire arm injuries (n=1;3.22%).

The risk factors for non-unions observed were obesity in 2(6.45%) patients and smoking among 3(9.67%) patients. As per AO classification system, there were 17(54.83%) patients with 32-D 5.2 fractures, 9(29.03%) patients with 32-D 4.2 fractures, 4(12.90%) patients with 32-D 5.1 fractures and 1(3.22%) patient with 32-D 4.1 fracture.

The duration since injury ranged between 9 to 24 months with a mean of 14.51±2.94 months. Among associated injuries encountered at the time of initial trauma there were 3(9.67%) patients with history of chest trauma, 2(6.45%) patients had sustained abdominal injuries and 1(3.22%) patient each had sustained moderate head injuries and trauma. These associated injuries were already managed elsewhere before presentation for the current procedure.

Before the current procedure, the patients had undergone 2-4 specific interventions for the fracture non unions. The mean of the procedures was 3.03± 0.795. Following debridement, the resultant bone gaps ranged from 3-5 cm. The mean was 4.00± 0.856 cm. Majority of the wounds (n=27;87.09%) had polymicrobial infection on tissue cultures. Staphylococcus aureus (n=28;90.32%) was the most frequent microbe grown. The other bacteria detected were Pseudomonas aeruginosa (n=14;45.16%), Methicillin Resistant Staphylococcus aureus (n=5;16.12%), Escherichia coli (n=5;16.12%), Klebsiella species (n=10;32.25%) and miscellaneous other bacterial species (n=3;9.67%).

According to ASAMI criteria, bone results are summarized in Table-I. The functional results are summarized in Table-II. Bone union was achieved among 28(90.32%) patients. (90.32% bone union rate). Infection was eradicated among 27(87.09%) patients. The bone transport time ranged from 33-155 days with a mean of 81.45±39.36 days.

**Table-I:** Bone results observed. (n=31).

	Results	Parameters	Number/ Percentage
1	Excellent	Infection eradication, bone union, deformities <7° and Limb length discrepancy <2.5 cm	8(%)
2	Good	Bone union + any two of the following: Infection eradication, <7° deformity, Limb length discrepancy <2.5 cm	19(%)
3	Fair	Bone union, persistent infection, <7° deformities and >2.5 cm limb length discrepancy	1(%)
4	Poor	Bone nonunion + infection + deformity >7° + Limb length discrepancy >2.5 cm	3(%)

**Table-II:** Functional results observed. (n=31).

	Results	Parameters	Number/ Percentage
1	Excellent	Active, no limp, minimal knee stiffness (loss of <15° knee extension), no Reflex sympathetic dystrophy, and significant pain	6(30%)
2	Good	Active, no limp, pain significant, no Reflex sympathetic dystrophy and 20° loss of knee extension	15(%)
3	Fair	Active, limping, knee stiffness, Reflex sympathetic dystrophy and significant pain	7(%)
4	Poor	Inactive and unable to return to daily activities	3(%)

The Plastic reconstructive procedures instituted included: local fasciocutaneous flaps (n=6;19.35%) and corrective surgeries for skin invagination between the bone ends (n=3;9.67%). The complications included: pin tract inflammation/ infection (n=25;80.64%), stiffness of knee joint (n=9;61.29%), K-wire loosening among (n=7;22.58%), skin invagination needing corrective plastic surgeries (n=3;9.67%), persistent non-union in (n=3;9.67%), unresolved infection (n=4;12.90%) and stiffness of hip joint (n=4;12.90%).

## DISCUSSION

Our study reflects the magnitude of the problem of infected non-unions among childhood and adolescent population. In our study relatively smaller number of (n=5;16.12%) patients had known risk factors for fracture non-unions in the form of obesity and active smoking. In the remaining majority of patients, other factors were responsible for the infected non-unions. These included complexity of the fractures, the associated soft tissue trauma, bacterial contamination of the wounds and lack of adequate initial debridement at the primary surgical interventions. The exact incidence of non-unions in the local population is not known, however the published studies reported femoral shaft fractures to account for 2% of all pediatric fractures.^2^,^3^

In this study we observed male predominance among our patients with femoral fractures. Our observation conforms to several published studies, where these fractures are mostly reported to be three times more common among boys than girls.^8^-^10^

In this study majority of the patients were over the age of 10 years. Our observation conforms to published studies. For instance, Kong H et al reported an average age of 10 (range of 6-15) years whereas Blasier RD et al reported 9-years as the average age at presentation. Some studies have reported a bimodal age distribution where the first peak incidence is encountered among children aged 1-3 years, and a second peak occurring during early adolescence.^7^-^10^

Majority of the patients in this study sustained the femoral fractures secondary to RTAs. Our finding conforms to several studies which have reported RTAs and falls from height as the commonest causes of the femoral fractures among their patients.^9^,^10^ None of our patients had the fractures secondary to child abuse or high energy sporting activity. Contrary to our observation, a good body of evidence from the Western populations suggests a significant contribution of child abuse and sports in causing these fractures among children and adolescents. In these populations about 80% of the isolated femoral shaft fractures among infants are attributed to child abuse.^9^,^11^,^12^

In this study we did not observe any sports related femoral fractures, however the published literature from the developed countries has reported such injuries. Adolescents who participate in sports such as the football and basketball are prone to sustain stress fractures of the femoral shaft and neck. If these remain undiagnosed and hence un-addressed, there is risk of their conversion into displaced fractures.^11^,^12^ Five patients in our study had known risk factors for developing non-unions. Three of them were regular smokers whereas two had morbid obesity. In the remaining majority of patients (83.87%) other factors that were responsible for the infected non-unions included complexity of the fractures, the associated soft tissue trauma, wound contamination at the time of injury and lack of proper initial debridement at the initial surgical interventions. Several published studies have suggested that the risk factors which result in fracture non unions among children and adolescents are very similar to the risk factors that lead to such fracture non-unions in the adult population. For instance, severe fractures (particularly the open and multi-fragmentary ones), obese patients, diabetes mellitus, active smoking, Vitamin-D deficiency, osteoporosis of any origin and osteoarthritis. The pediatric population has certain additional predisposing factors for non-unions too. For example, age ≥11 years, use of various pharmacological agents such as the bisphosphonate (employed for a variety of metabolic bone disorders among children), glucocorticoids, anticoagulant therapy or opioid.^13^,^14^

The infected non unions of femur necessitate a radical surgical approach that entails through debridement of the dead and necrotic bone ends. The resultant bone defect can be reconstructed in a number of ways. For instance, use of bone grafts or vascularized bone flaps. The Masquelet technique of staged membrane-induction may also be employed. These procedures have their own attended disadvantages. For instance, donor site morbidity, poor graft take, risk of flap failure, possibility of re-fracture and the need for several staged procedures.^9^,^15^

Among our patients we employed the Ilizarov technique of segmental bone transport. It offered a variety of added benefits for the patient as well as the operating surgeon. These included the use of limited surgical access, reduced operating time, lesser surgical dissection and minimal blood loss. These factors ensured added safety given the young age and hemodynamic fragility of the children and adolescents. The fixator was strong and stable enough to allow early ambulation as well as immediate weight bearing in the postoperative phase. Our abovementioned observations in are in conformity with several previously published studies.^5^,^6^,^16^,^17^

### Strengths and Limitations of the study

The current study has certain strengths as well as presents some limitations. The study has attempted to document the local epidemiological data regarding presentation and outcome of femoral infected non unions in children and adolescents. This evidence base provides crucial insights about the problem and hence future directions for evolving preventive strategies. One limitation of the study is its being a qualitative research rather than being a detailed quantitative analysis of the various factors responsible for causing infected fracture non unions in the given population. Secondly there was relatively small volume of cases treated over a period of five years. We recommend future studies to confirm upon our findings and improve upon the aforementioned limitations.

## CONCLUSION

Majority of the patients were males and aged 9-16 years. Road traffic accidents were the leading cause of the fractures. The various risk factors for infected fracture non-unions included obesity, smoking, complexity of the fractures, the associated soft tissue trauma, bacterial contamination of the wounds and lack of adequate debridement at the primary surgical interventions. The Ilizarov method of segmental bone transport was effective in treating the majority of infected non-unions. Primary prevention with religious wound debridement at the primary surgery would help to reduce the burden of these cases.

### Authors’ Contribution:

**MS & FUKZ:** Designed the study and wrote the manuscript.

**KB, FUKZ, EM & AUR:** Performed data collection and analysed the results.

**FUKZ:** Responsible for accuracy and integrity of the work.

All authors have approved the manuscript.
